# EGFR and FGFR Pathways Have Distinct Roles in Drosophila Mushroom Body Development and Ethanol-Induced Behavior

**DOI:** 10.1371/journal.pone.0087714

**Published:** 2014-01-31

**Authors:** Ian F. G. King, Mark Eddison, Karla R. Kaun, Ulrike Heberlein

**Affiliations:** Department of Anatomy, Univeristy of California San Francisco, San Francisco, California, United States of America; University of Houston, United States of America

## Abstract

Epidermal Growth Factor Receptor (EGFR) signaling has a conserved role in ethanol-induced behavior in flies and mice, affecting ethanol-induced sedation in both species. However it is not known what other effects EGFR signaling may have on ethanol-induced behavior, or what roles other Receptor Tyrosine Kinase (RTK) pathways may play in ethanol induced behaviors. We examined the effects of both the EGFR and Fibroblast Growth Factor Receptor (FGFR) RTK signaling pathways on ethanol-induced enhancement of locomotion, a behavior distinct from sedation that may be associated with the rewarding effects of ethanol. We find that both EGFR and FGFR genes influence ethanol-induced locomotion, though their effects are opposite – EGFR signaling suppresses this behavior, while FGFR signaling promotes it. EGFR signaling affects development of the Drosophila mushroom bodies in conjunction with the JNK MAP kinase *basket (bsk)*, and with the Ste20 kinase *tao,* and we hypothesize that the EGFR pathway affects ethanol-induced locomotion through its effects on neuronal development. We find, however, that FGFR signaling most likely affects ethanol-induced behavior through a different mechanism, possibly through acute action in adult neurons.

## Introduction

Though alcohol dependence is highly influenced by genetics, it has proven difficult to conclusively identify genes that confer risk or that could be targets for therapy [1]–[3]. Animal models, including *Drosophila melanogaster*, are an important tool for the identification of genes that influence the behavioral response to ethanol [4]–[7]. Drosophila inhabit environments rich in ethanol, and are highly adapted to ethanol exposure [8]. Because genes controlling neuronal development and function are well conserved between Drosophila and mammals, and because flies exhibit a number of ethanol-induced behaviors that are analogous to mammalian behaviors, Drosophila has been used successfully to identify genetic pathways affecting ethanol-induced behaviors that have conserved roles in mammalian systems [9]–[12].

Ethanol has both stimulant and sedative effects in Drosophila [4], [13]. High doses of ethanol vapor induce loss of postural control and sedation, analogous to its sedative effects of ethanol on mammals [14] . However, moderate doses of ethanol have a stimulant effect on flies, causing a strong, sustained increase in locomotion. This ethanol-induced locomotion is analogous to locomotor stimulation seen in mammals, occurring at similar internal ethanol concentrations [14]. Significantly, in Drosophila, as in mammals, doses of ethanol that induce increased locomotor activity are associated with the rewarding effects of intoxication [6], [15].

EGFR and FGFR signaling mediate numerous biological processes in Drosophila and mammals. In flies, signaling through a single EGFR (*Egfr*) affects cell fate, proliferation, migration, and survival at multiple points in development [16]. *Egfr* has many roles in the development of the nervous system, controlling neuronal organization, inducing cell fate decisions and differentiation [17]–[20], and serving as a gliotrophic factor [21]. The Drosophila genome has two FGFR genes, *breathless* (*btl*) and *heartless* (*htl*). *btl* has been best characterized in Drosophila as a factor controlling branching morphogenesis during the development of the tracheal system [22], while *htl* was identified by its effects on cell migration in the developing mesoderm [23]. However, both are active in the developing Drosophila nervous system, promoting the outgrowth and guidance of axons in the developing brain and peripheral nervous system [24], [25]. EGFR and FGFR activate common downstream signaling components [26], and in Drosophila act together in a number of developmental processes [27]–[29], including an overlapping role in the guidance of developing sensory axons [25].

EGFR pathway activity antagonizes the sedative effects of ethanol in flies. Mutations in the Ste20 kinase *happyhour* (*hppy*) increase EGFR signaling in the central nervous system and lead to sedation resistance, while mutations that result in reduced EGFR signaling cause sedation sensitivity [10]. Similarly, loss of function mutations in *arouser* (*aru*), a homologue of the mammalian EGFR substrate *EPS8* [30], cause ethanol-sedation sensitivity and result in an increase in the number of synaptic terminals in the adult brain [31]. Increased synapse number and ethanol sensitivity in *aru* mutants are established during development, but can be restored by social isolation in the adult fly [31]. EGFR signaling also mediates the sedative response to ethanol in mice, suggesting its role in behavior is conserved in mammals [10].

Mutations in *tao*, a protein kinase of the Ste20 family, have a profound effect on ethanol-stimulated behavior in Drosophila, virtually eliminating ethanol-induced locomotion [32]. *tao* function is required for the normal development of the central brain, particularly in axon pathfinding in the developing mushroom bodies (MB) [32], though the disruption of the MB is not required for the behavioral phenotype of *tao^EP1455^* [33]. In MB development *tao* interacts negatively with both the kinase *par-1* and the JNK MAP kinase *basket*, and through *par-1* regulates the phosphorylation state of the microtubule-associated protein Tau [32], [33].

Tao family kinases interact genetically with RTK signaling genes, both in flies and in mammals, negatively regulating RTK signaling in both systems. Overexpression of Tao in the Drosophila eye can suppress a rough-eye phenotype caused by constitutively activated RTKs including *Egfr*, *btl* and *htl* [34]. In mammalian cells, stimulation with Epidermal Growth Factor (EGF) decreases the activity of the Tao homologue Taok3, and Taok3 in turn suppresses EGF-induced activation of JNK MAP kinase signaling [35].

To investigate whether RTK signaling genes are important for ethanol-induced behavior in Drosophila, we tested the effects of EGFR and FGFR signaling pathway mutations on ethanol-induced locomotor stimulation. We found that mutations in EGFR pathway genes increase ethanol-induced locomotion, and also suppresses the behavioral phenotype of a *tao* mutant, *tao^EP1455^.* EGFR pathway mutations can also suppress the defect in MB development caused by *tao^EP1455^*, indicating that EGFR and *tao* have antagonistic functions in the development of MB. Suppression of the *tao* mutant phenotype by EGFR pathway mutations is enhanced by a mutation in the JNK MAP kinase *basket* (*bsk*), consistent with these genes acting in a common pathway. Further, we show that the FGFR gene *htl* also mediates ethanol-induced behavior in flies. Interestingly, we find that acute overexpression of Htl in the adult nervous system can affect ethanol-induced hyperactivity, suggesting that FGFR signaling may influence this behavior through a different mechanism than EGFR signaling.

## Results

### EGFR Pathway Genes Affect Ethanol-induced Locomotion

**Figure 1:**
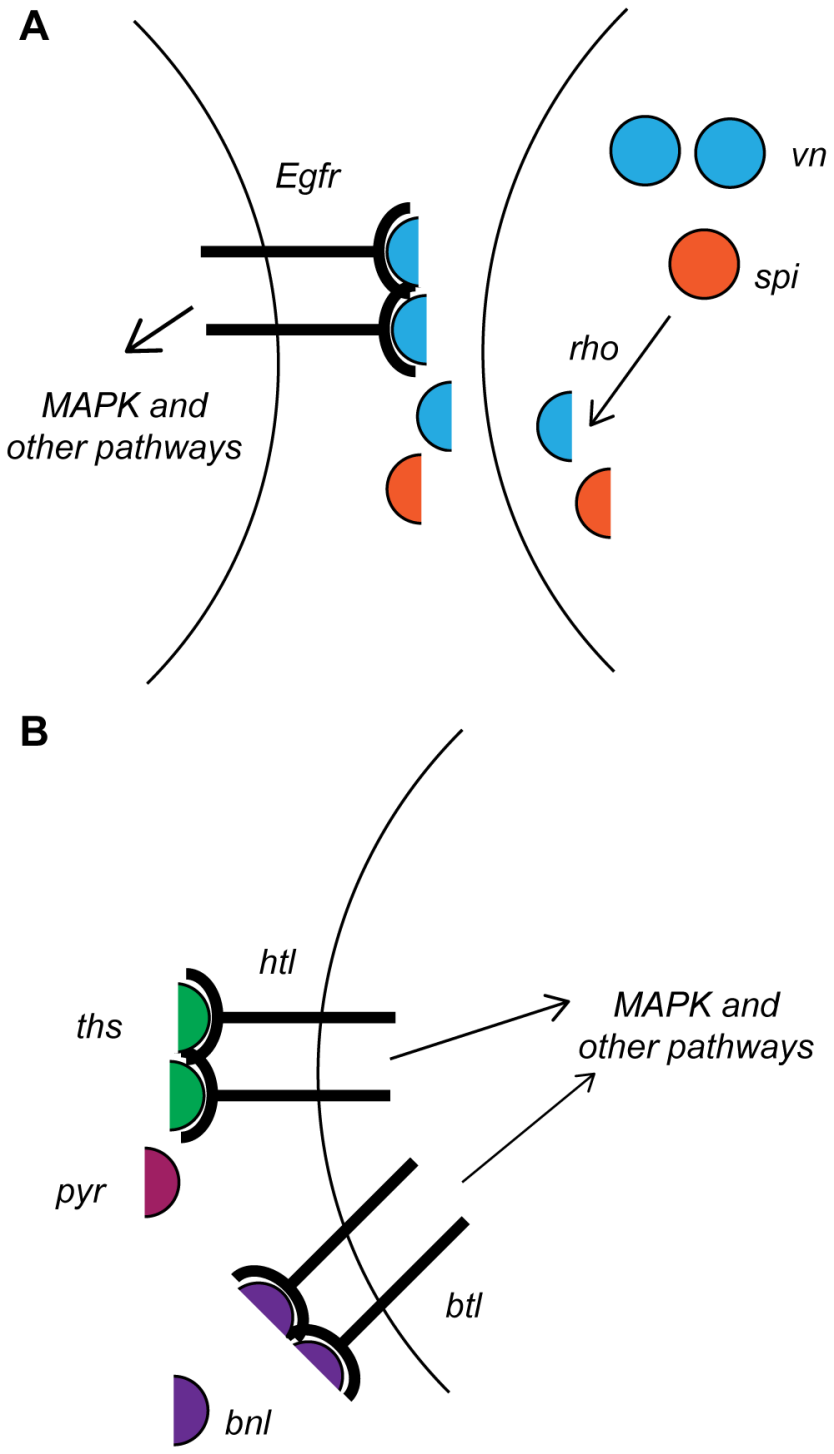
Drosophila EGFR and FGFR signaling pathways. (A) Schematic of EGFR pathway genes in this study. The EGFR ligands Vein (vn) and Spitz (spi) are synthesized as precursor peptides, which are cleaved to an active form by the protease Rhomboid (rho). These processed ligands are then secreted, and bind the receptor, Egfr. Bound receptor dimers initiate signaling that can activate a number of downstream pathways, most notably ERK and JNK MAP kinase cascades. (B) Schematic of FGFR pathway genes. Two FGFR receptors, Breathless (btl) and Heartless (htl) are activated by separate ligands – Heartless by Pyramus (pyr) or Thysbee (ths), and Breathless by Branchless (bnl). FGFR activation can also initiate multiple downstream signaling pathways, including MAPK cascades.

**Figure 2:**
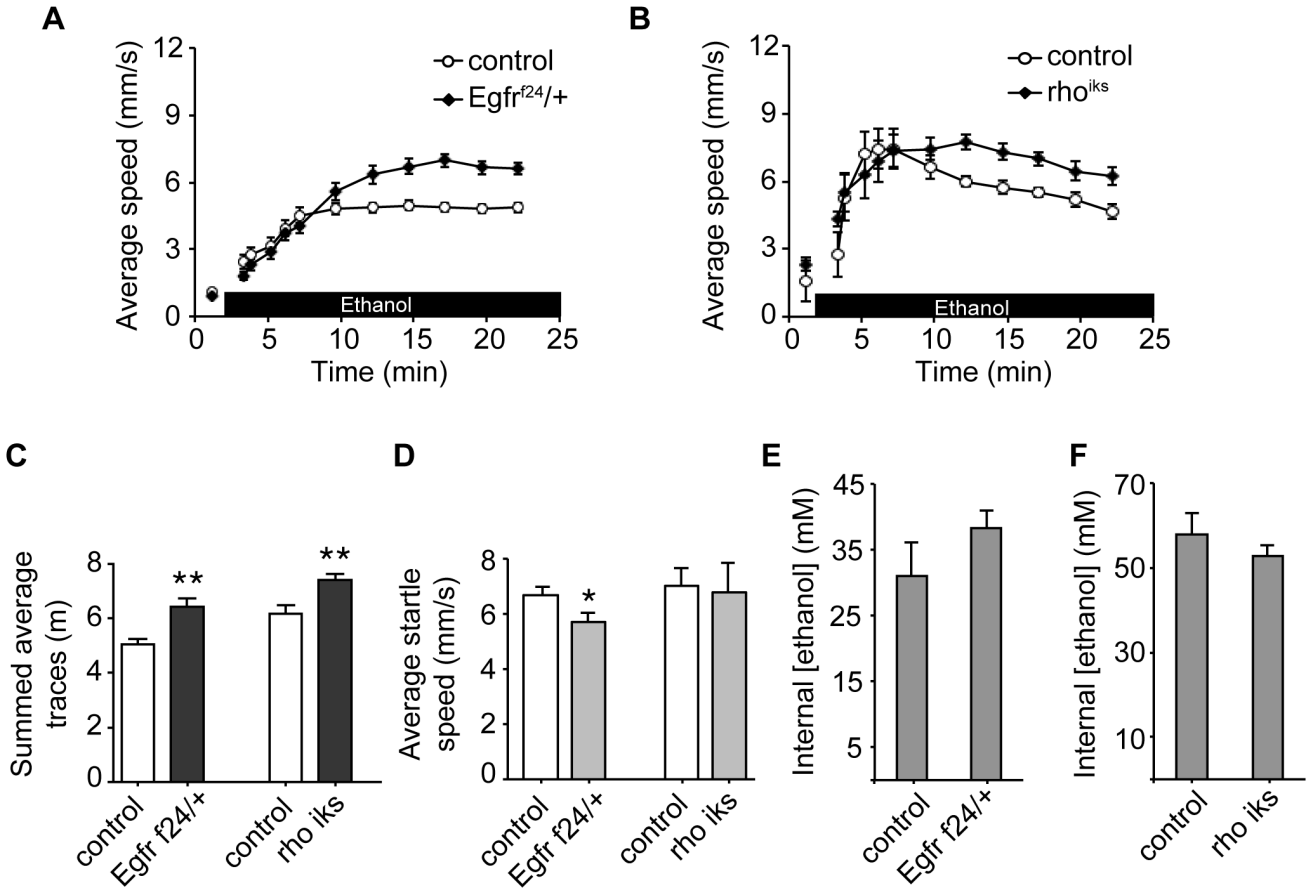
Mutations in *Egfr* and *rho* increase ethanol-induced locomotion. Ethanol-induced locomotion of (A) *Egfr^f24^/+* and (B) *rho^iks^*, compared to controls. First point represents baseline locomotion before ethanol exposure. (C) Area-under-curve quantification of (A) and (B). (D) Peak locomotion during ethanol-induced olfactory startle for mutant and control strains. (E) Ethanol absorption for *Egfr^f24^/+*, compared to wild-type controls. (F) Ethanol absorption after 15 minutes for *rho^iks^* flies, compared to wild-type controls. *p<0.05, **p<0.01, Student’s T-test.

Because of previous evidence suggesting an interaction between *tao* and RTK signaling genes in Drosophila [34], we investigated whether RTK genes might also affect ethanol-induced locomotor stimulation. We first tested flies with mutations in *Egfr* and upstream effectors of EGFR signaling (Fig. 1A). We found that flies heterozygous for an *Egfr* null allele (*Egfr^f24^/+*) showed increased ethanol-induced locomotion (Fig. 2A,C), and that a hypomorphic mutation in *rhomboid* (*rho^iks^*), a protease required for EGFR ligand processing (Fig. 1A) [36], also increased ethanol-induced locomotion (Fig. 2B,C). To assess whether these mutants had defects in locomotion that might confound the results of the ethanol-induced locomotion assay we measured peak locomotor speed during the olfactory startle response upon first exposure to ethanol [14]. *rho^iks^* flies showed normal startle speed, and *Egfr^f24^/+* flies had a slightly decreased startle speed (Fig. 2D), indicating that neither line exhibits hyperlocomotion that might explain their ethanol-response phenotype. Both lines also had normal low baseline locomotion in the absence of ethanol (Fig. 2A,B), and had normal levels of ethanol absorption (Fig. 2E,F).

**Figure 3:**
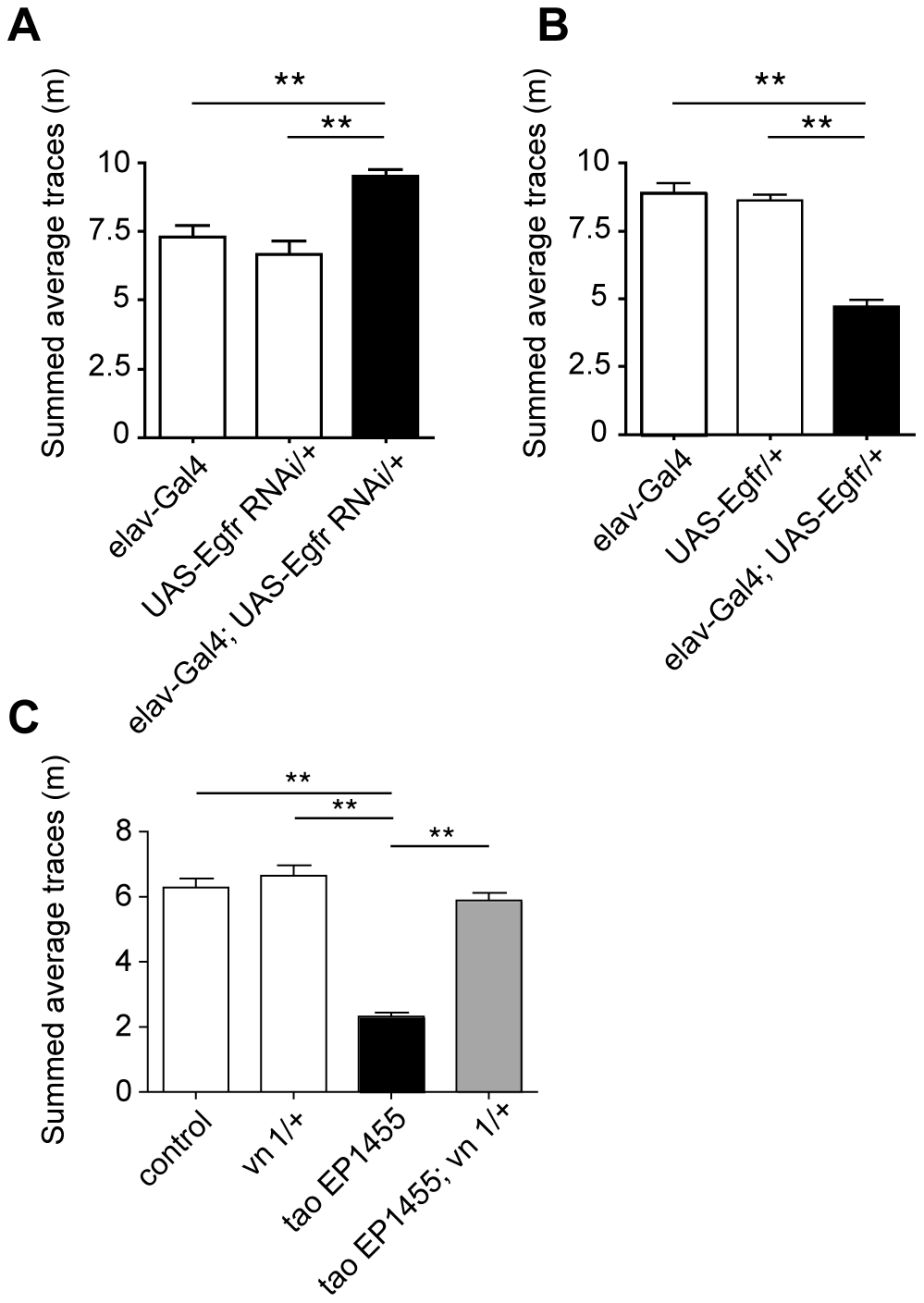
Egfr expression level in neurons affects ethanol-induced locomotion. (A) Ethanol induced locomotion of flies overexpressing wild-type Egfr in neurons, under the control of *elav-Gal4*, compared to single transgenic controls. (B) Ethanol-induced locomotion of flies with RNAi-mediated knockdown of *Egfr* expression in neurons, compared to single transgenic controls. (C) Ethanol-induced locomotion of *tao^EP1455^; vn^1^/+* flies, compared to single mutants and wild-type control. **p<0.01, One-way ANOVA with Neuman-Keuls post-hoc test.

We used the Gal4-UAS expression system [37] to establish whether EGFR signaling in neurons affects ethanol-induced locomotion. We crossed the *elav^C155^-Gal4* driver line, which expresses Gal4 exclusively in neurons [38], to lines carrying transgenes for Gal4-dependent RNAi knockdown (*UAS-EgfrRNAi*) [10] or overexpression of wild type Egfr (*UAS-Egfr*) [39]. Compared to single transgenic controls, knockdown of *Egfr* in neurons increased ethanol-induced locomotion (Fig. 3A), consistent with the effects of EGFR pathway loss-of-function mutations. Conversely, overexpression of Egfr in neurons reduced ethanol-induced locomotion dramatically (Fig. 3B). These experiments suggest that levels of EGFR signaling in neurons influence the stimulatory effects of ethanol in Drosophila.

Overexpression of Tao can suppress defects in eye development caused by EGFR overexpression, suggesting that these genes might function antagonistically in neurodevelopment [34]. To investigate whether this relationship also holds for ethanol-induced behavior, we tested whether a mutation in the EGFR pathway could suppress the ethanol-induced locomotion deficit of *tao^EP1455^*. We combined *tao^EP1455^* with a heterozygous hypomorphic mutation in the EGFR ligand *vein* (*vn^1^*), an activator of EGFR signaling during the development of the embryonic nervous system (Fig. 1A) [40]. In contrast to *tao^EP1455^* flies, which showed severely reduced ethanol-induced locomotion, *tao^EP1455^*; *vn^1^*/+ flies had normal ethanol-induced locomotion (Fig. 3C). *vn^1^/+* heterozygotes had normal ethanol-induced locomotion, implying that the recovery seen in the double mutant is not likely to be due to separate additive effects of these mutations. Instead, these results suggest that neurodevelopmental defects that reduce ethanol-induced locomotion in *tao^EP1455^* can be suppressed by reduced EGFR signaling.

### EGFR and JNK Pathway Mutations Together Restore Mushroom Body Morphology in *tao* Mutants

**Figure 4:**
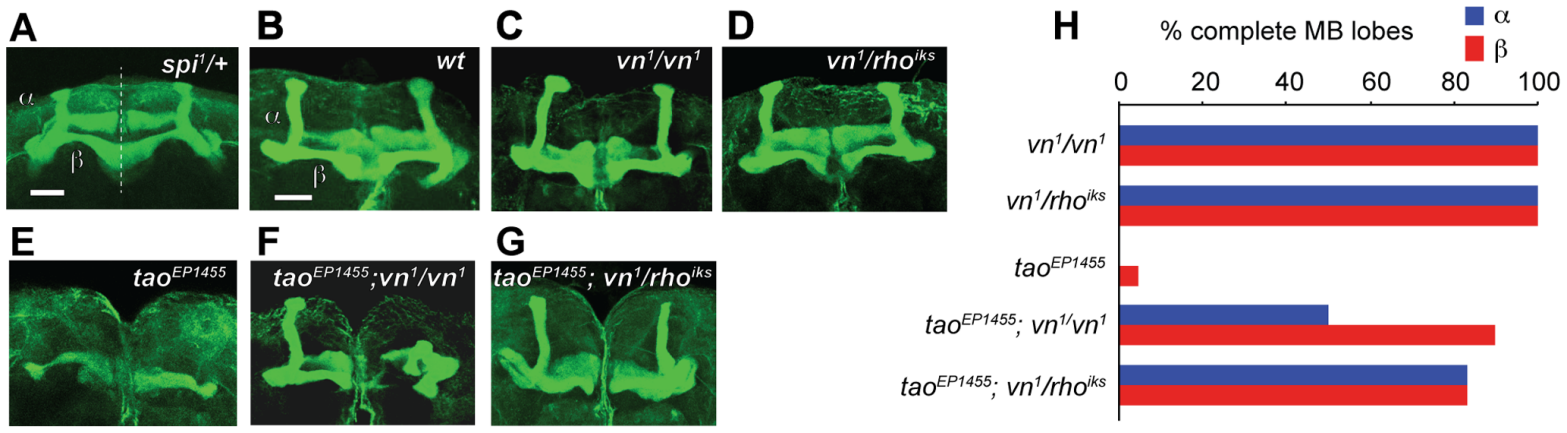
EGFR pathway mutations affect mushroom body development. (A) Anti-Fas II staining of wholemount brain from *spi^1^/+*. Dashed line represents the midline. Scale bar = 10 µm. (B–H) Suppression of *tao^EP1455^* MB lobe morphology phenotype by mutations in *vn* and *rho*. Scale bar = 10 µm. (B*) wt*, (C) *vn^1^/vn^1^* and (D) *vn^1^/rho^iks^* brains have normal MB lobes. (E) *tao^EP1455^* lacks MB α and β lobes. MB lobes in brains of (F) *tao^EP1455^;vn^1^/vn^1^* and (G) *tao^EP1455^;vn^1^/rho^iks^* flies. (H) Quantification of the proportion of normal MB lobes for genotypes in (C–G).

The EGFR pathway is required during development for normal ethanol-induced sedation [31]. Because of hypothesized interactions with *tao*, we sought to determine whether EGFR signaling is required in the development of the Drosophila brain. Because *tao* mutations cause defects in central brain development, and in particular, can strongly impair the formation of adult MB axon lobes [32], we hypothesized that EGFR pathway mutations might also affect adult MB development. We examined adult MB morphology in EGFR pathway mutants by immunostaining with anti-Fasciclin II (FasII), which stains the MB α/β and γ axon lobes [41]. Interestingly, we found that flies heterozygous for a mutation in the strong Egfr-activating ligand *spitz* (*spi^1^*) (Fig. 1A) frequently had MB β lobes that fused across the midline (7 of 12 brains examined) (Fig. 4A), an indication of axon overgrowth [42].

**Figure 5:**
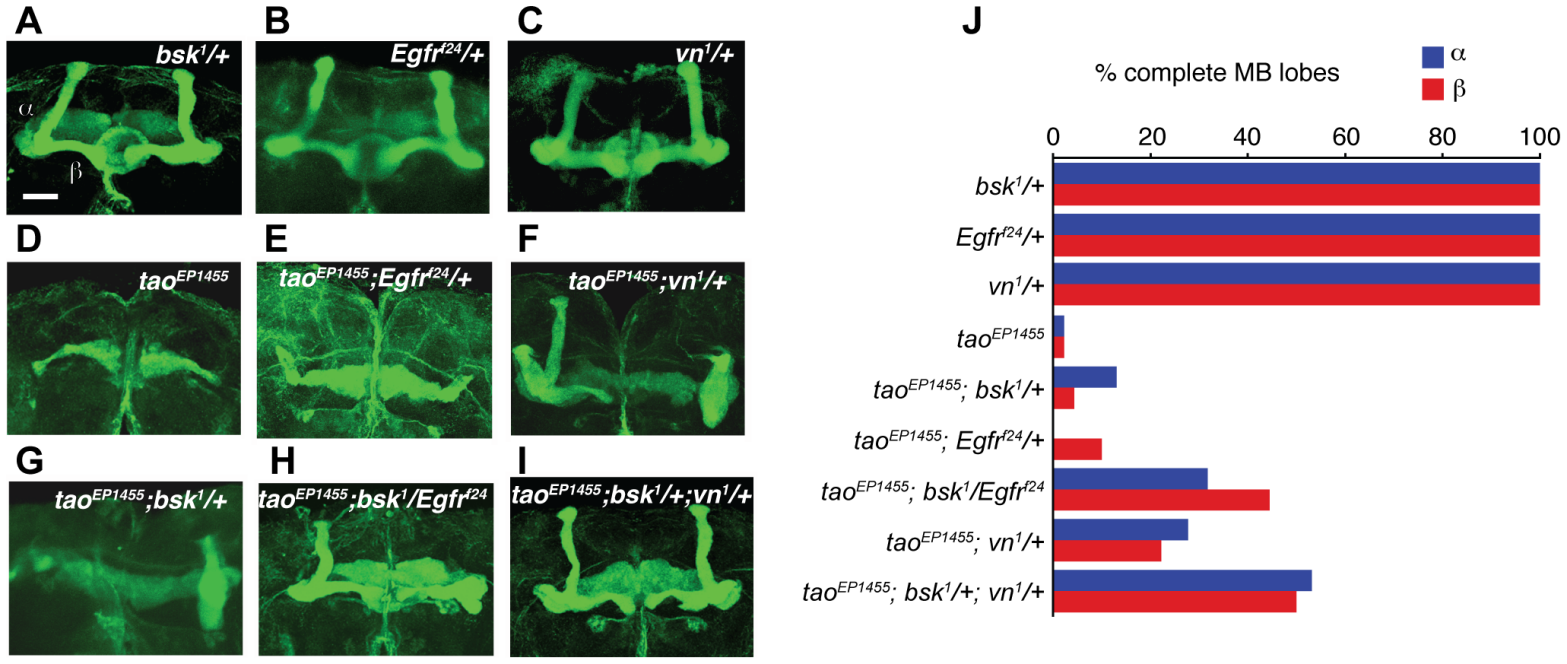
EGFR and JNK pathway components interact to affect mushroom body development. Anti-Fas II staining of wholemount brains from (A) *bsk^1^/+*, (B) *Egfr^f24^/+*, (C) *vn^1^/+*, (D) *tao^EP1455^*, (E) *tao^EP1455^;Egfr^f24^/+*, (F) *tao^EP1455^;vn^1^/+*, (G) *tao^EP1455^;bsk^1^/+*, (H) *tao^EP1455^;bsk^1^/Egfr^f24^*, (I) *tao^EP1455^;bsk^1^/+;vn^1^/+.* Scale bar = 10 µm. (J) Quantification of the proportion of normal MB lobes for genotypes in (A–I).

The β lobe overextension phenotype of *spi^1^/+* suggests that EGFR signaling might act as a positive signal for MB axon extension, and might be antagonistic to *tao* mutations in MB development. Flies carrying the *tao^EP1455^* P-element insertion mutation have severe defects in the formation of the MB axon lobes [32]. To test whether EGFR pathway mutations could suppress the MB development defects seen in *tao^EP1455^*, we examined MB morphology in *tao^EP1455^*; *vn^1^/+* double mutants. As reported previously, *tao^EP1455^* flies lack MB α/β lobes (Fig. 4B,E,H. Fig. 5D,J). In contrast, *vn^1^/+* heterozygotes had normal MB lobes (Fig. 5C,J). The double mutant, *tao^EP1455^*; *vn^1^/+* had a modest recovery of normal MB α/βlobes (Fig. 5F,J). The degree of recovery does not match the full recovery of the ethanol-induced behavioral phenotype that we observe in *tao^EP1455^*; *vn^1^/+* (Fig. 3C). It is possible that this partial recovery of MB morphology is sufficient to restore normal behavior to *tao^EP1455^.* However, as we have observed previously, the behavioral phenotype of *tao^EP1455^* can be suppressed by mutations which have no apparent effect on its MB morphology defects [33], implying that *tao* and the EGFR pathway also influence other loci outside of the MB that are required for normal ethanol-induced behavior.

To determine if EGFR signaling acts at other loci that have been implicated in ethanol-induced locomotor stimulation, we overexpressed Egfr under the control of Gal4 driver lines expressing in known loci controlling ethanol-induced locomotion. We expressed Egfr in dopaminergic neurons using TH-Gal4 [43], and in ellipsoid body (EB) R2/R4 neurons using 11.148-Gal4 [43]. Overexpression of Egfr using either Gal4 line did not alter levels of ethanol-induced locomotion from that found in single transgenic controls (data not shown), indicating that EGFR signaling acts in as yet unidentified components of the circuitry governing ethanol-induced locomotion.

Stronger allele combinations (*vn^1^/vn^1^* and *vn^1^*/*rho^iks^*) were able to suppress the MB development phenotype of *tao^EP1455^* more completely. Both *vn^1^/vn^1^* and *vn^1^*/*rho^iks^* flies had normal MB morphology (Fig. 4B-D,H), and we frequently observed normal MB α/β lobes in the brains of *tao^EP1455^; vn^1^/vn^1^*, and *tao^EP1455^; vn^1^*/*rho^iks^* adults (Fig. 4F–H). Together, these experiments show that mutations affecting EGFR signaling can restore proper formation of MB axon lobes in a *tao* mutant.

Others have shown that the JNK pathway controls extension of MB axons during the development of the MB lobes [42], and we have shown previously that JNK pathway mutations suppress the MB development phenotype of *tao^EP1455^*, suggesting that *tao* negatively regulates JNK signaling in extending MB axons [33]. Because we had observed interactions between EGFR pathway components and *tao* in MB development, we hypothesized that EGFR, *tao,* and JNK signaling might act together to direct MB axon lobe formation. We first tested whether single weak mutations in either *Egfr* or in *bsk*, the lone JNK MAP kinase in Drosophila, could enhance recovery of MB α/β lobes in *tao^EP1455^*. Flies heterozygous for *bsk^1^* had normal MB morphology, and *bsk^1^/+* caused very little recovery of MB α/β lobes in a *tao^EP1455^* background (Fig. 5A,D,G,J). Similarly, heterozygous mutations in EGFR pathway genes (*Egfr^f24^*/+, *vn^1^/+*) had normal MB morphology and produced very little recovery of MB lobes in a *tao^EP1455^* background (Fig. 5B–F,J).

In contrast, when we examined triple mutants that combined *tao^EP1455^* with both *bsk* and EGFR pathway mutations together, we observed considerable recovery of normal MB α/β lobes (Fig. 5H–J). Recovery in *tao^EP1455^;bsk^1^/Egfr^f24^* was greater than the additive effects of the single mutations (Fig. 5H,J), suggesting that *Egfr* and *bsk* may function in a common pathway in MB development. We observed similar MB lobe recovery in flies with mutations in *tao*, *bsk* and *vn*: *tao^EP1455^;bsk^1^/+;vn^1^/+* showed considerable recovery of α/β lobes (Fig. 5I,J) compared to recovery in *bsk^1^/+* and *vn^1^/+* mutants alone (Fig. 5F,G,J), consistent with a genetic interaction between *tao*, *bsk* and *vn*.

Together, the results of this study suggest that the EGFR signaling pathway acts in conjunction with *tao* and with JNK MAPK signaling in the development of the adult brain, likely affecting the development of loci required for ethanol-induced locomotion.

### The FGFR gene *htl* Mediates Ethanol-induced Behavior

**Figure 6:**
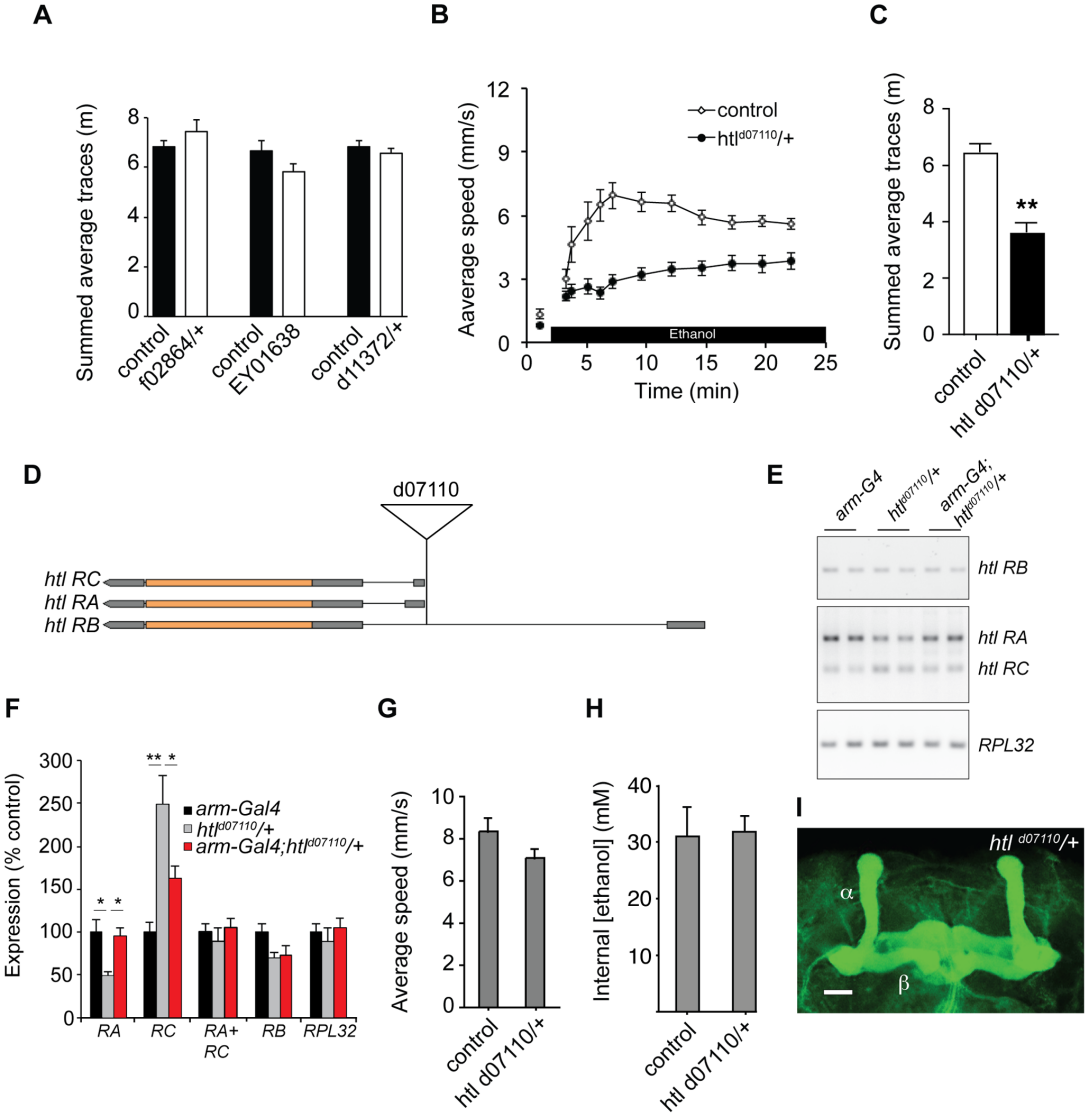
A mutation in the FGFR gene *htl* reduces ethanol-induced locomotion. (A) Area-under-curve quantification of ethanol-induced locomotion for three lines with P-element insertions in the FGFR gene *btl* (f0286/+, EY01638, d11372/+) versus controls. (B) Ethanol-induced locomotion assay for *htl^d07110^/+*, compared to controls. (C) Area-under-curve quantification of (B) **p<0.01 Student’s T-test. (D) Schematic of the transcripts produced by the *htl* gene, adapted from Flybase (flybase.org). The locus produces three transcripts, RA, RB and RC. The d07110 line has an XP element inserted upstream of the start site for the RA and RC isoforms. (E) RT-PCR of *htl* transcripts. Total RNA was isolated from heads of *armadillo-Gal4 (arm-Gal4), htl^d07110^/+* and *arm-Gal4; htl^d07110^*/+ flies. RT-PCR was performed with primers amplifying the RB transcript, or the RA and RC transcripts, and primers for a housekeeping gene control (*RPL32*). (F) Quantification of RT-PCR experiments. Levels of *htl RA*, *RB* and *RC* transcripts, and the combined level of *RA* and *RC* expression (*RA+RC*) as percent of expression in the *armadillo-Gal4* control line. Error bars represent SEM. *p<0.05, **p<0.01, One-way ANOVA with Neuman-Keuls post-hoc test. *n* = 3. (G) Peak locomotion during ethanol-induced olfactory startle for mutant and control strains. (H) Ethanol absorption after 20 minutes for *htl^d07110^/+* flies compared to wild-type controls. (I) Anti-Fas II staining of wholemount brain from *htl^d07110^/+*. Scale bar = 10 µM.

Because *tao* can also negatively regulate FGFR signaling during development, we also tested whether mutations in *btl* and *htl*, the two FGFR receptor genes in Drosophila, could affect ethanol-induced locomotion (Fig. 1B). We tested two lethal P-element insertions in *btl* as heterozygotes, and one viable insertion as a homozygote, but found no effects on behavior (Fig. 6A). However, flies heterozygous for *htl^d07110^*, a lethal P-element insertion in *htl*, showed reduced ethanol-induced locomotion (Fig. 6B,C), raising the possibility that *htl* could affect ethanol-induced behavior. *htl^d07110^* fails to complement the strong loss of function allele *htl^AB42^* for lethality, indicating that it disrupts *htl* expression (data not shown). Consistent with this, RT-PCR analysis of RNA extracted from heads of *htl^d07110^*/+ flies showed that levels of the *htl RA* transcript were reduced in comparison to controls (Fig. 6D-F). Interestingly, levels of the *RC* transcript were increased in the mutant, and as a result the total level of *RA* and *RC* expression was not significantly changed in the mutant. (Fig. 6E,F). Levels of the *RB* transcript and of the housekeeping gene *RPL32* were also not significantly different between the mutant and the control (Fig. 6E,F). Because *htl^d07110^* is non-viable in combination with the loss-of-function allele *htl^AB42^*, these results suggest that the behavioral phenotype seen in *htl^d07110^/+* flies might arise specifically from a deficit in expression of the *RA* trasnscript. *htl^d07110^/+*flies had a normal olfactory startle response and baseline locomotion (Fig. 6B,G), absorbed ethanol normally (Fig. 6H), and had normal gross brain morphology (data not shown) and mushroom body morphology (Fig. 6I).

**Figure 7:**
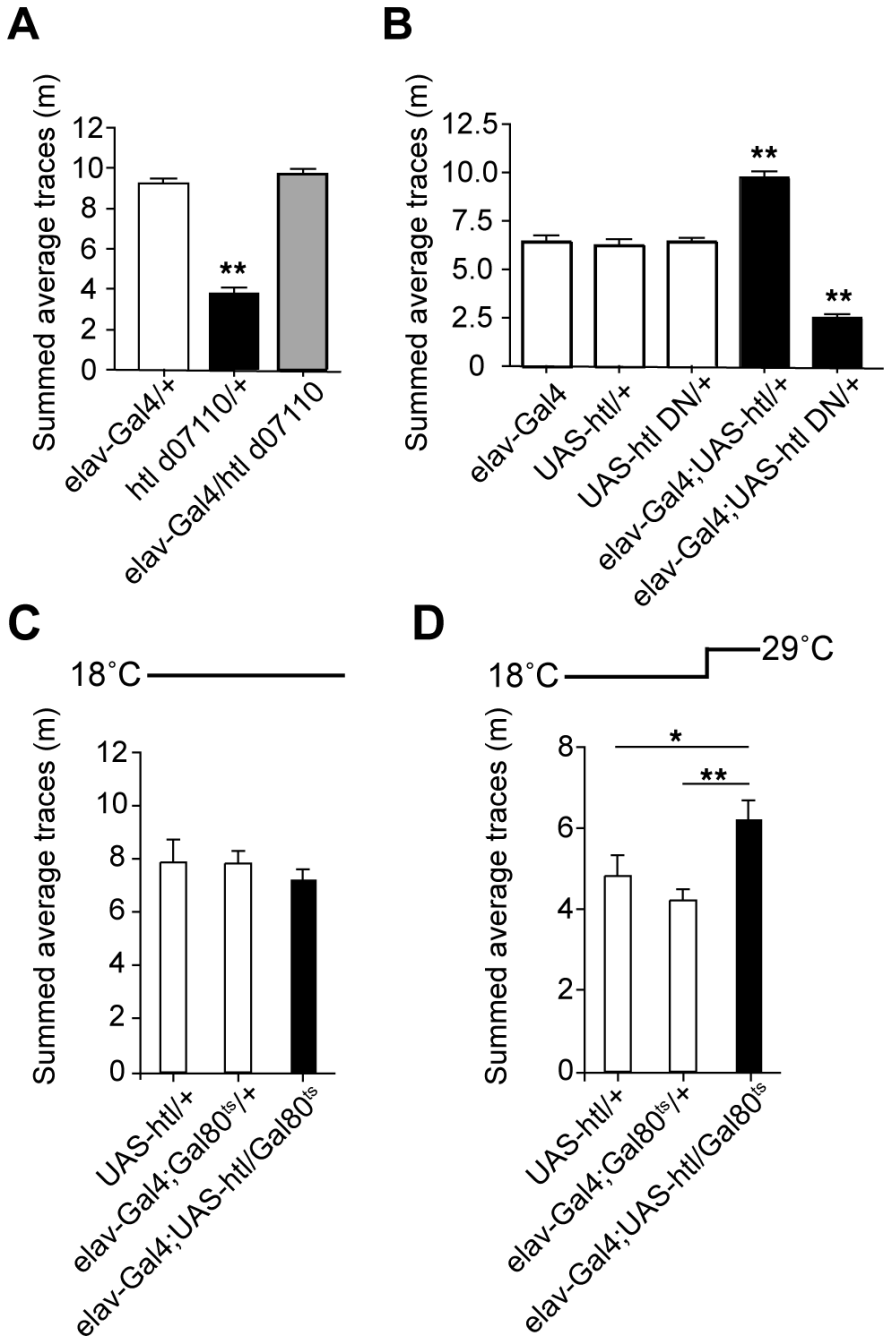
*htl* is required in neurons for normal ethanol-induced locomotion. (A) Neuronal-specific rescue of the behavioral phenotype of *htl^d07110^/+.* Area-under-curve quantification of ethanol-induced locomotion for *elav^3E1^-Gal4/htl^d07110^*, compared to single transgenic controls. (B) Ethanol-induced locomotion of flies overexpressing wild-type or dominant-negative Htl under the control of *elav^C155^-Gal4*, compared to single transgenic controls. (C,D) Overexpression of Htl in the adult nervous system increases ethanol-induced hyperactivity. Area-under-curve quantification of ethanol-induced locomotion for flies expressing Htl under the control of *elav^C155^-Gal4*. Expression of *UAS-htl* by *elav-Gal4* was placed under the control of *Gal80^ts^*. (C) Flies maintained at 18°C, at which Gal80^ts^ represses Gal4 function. (D) Flies raised at 18°C, then shifted to 29°C (at which Gal80^ts^ is inactivated) for 48 hours before behavioral assays. *p<0.05, **p<0.01 One-way ANOVA with Neuman-Keuls post-hoc test.

We used the Gal4-UAS system to test whether the reduced ethanol-stimulated locomotion seen in *htl^d07110^/+* flies was due to the loss of *htl* expression in neurons, The XP element in *htl^d07110^* has UAS sequences that allow Gal4-dependent expression of the RA and RC isoforms of *htl* [44]. Expression of both the *RA* and *RC* transcripts was restored to near normal levels when *htl^d07110^* was combined with *armadillo*-*Gal4*, a driver line that expresses strongly in the adult brain [45] (Fig. 6E,F). To determine if restoring *htl* expression in all neurons could rescue the behavioral phenotype of *htl^d07110^/+* mutants, we measured ethanol-induced locomotion of *htl^d07110^/+* combined with the weaker pan-neuronal driver *elav^3E1^-Gal4*. Gal4-driven expression of *htl* in neurons was able to rescue the ethanol-induced locomotion defect of *htl^d07110^/+* flies, suggesting that *htl* is required in neurons for normal ethanol-induced behavior (Fig. 7A). To test whether altering levels of *htl* activity in neurons could affect ethanol-induced locomotion, we used *elav^C155^-Gal4* in combination with UAS transgenes to overexpress wild-type (Htl^wt^) and dominant negative (Htl^DN^) forms of Htl in neurons [46]. Overexpression of Htl^wt^ increased ethanol-induced locomotion, while expression of Htl^DN^ in neurons decreased it (Fig. 7B), suggesting that levels of Htl activity in neurons regulate ethanol-induced locomotion.

Because we could discern no effects of *htl* mutations on brain morphology, we tested whether changing Htl levels in the adult nervous system might affect ethanol-induced behavior. We used the *Gal80^ts^* (TARGET) system for temporal control of Gal4-dependent expression to overexpress Htl^wt^ acutely in the nervous system of the adult [47]. No difference was seen between flies carrying both *UAS-htl* and *elav-Gal4* and controls when raised at 18°C, the permissive temperature for Gal80^ts^, at which the activity of Gal4 is inhibited (Fig. 7C) However, when flies were raised at 18°C, then shifted to 29°C after eclosion, inactivating Gal80^ts^ and allowing Gal4-dependent *htl* expression in the adult nervous system, we observed increased ethanol-induced locomotion (Fig. 7D), consistent with a role for *htl* in the acute function of the adult nervous system. Thus signaling through the FGFR *htl* is likely to affect ethanol-induced behavior through a different mechanism from the EGFR pathway, in spite of their shared downstream effectors and their many shared developmental functions.

## Discussion

We have shown that in Drosophila, EGFR signaling in neurons can affect ethanol-induced locomotion, a behavior that correlates to the rewarding effects of ethanol. Loss of function mutations in EGFR pathway genes resulted in increased ethanol-induced locomotion and overexpression of Egfr resulted in reduced ethanol-induced locomotion, suggesting that the EGFR pathway inhibits this behavior. We have also uncovered a novel role for the FGFR gene *htl* in ethanol-induced behavior. In contrast to mutations in the EGFR pathway, loss of the FGFR *htl* in neurons reduces ethanol-stimulated locomotion, suggesting that *htl* promotes this behavior.

While the EGFR and FGFR pathways use common components and can act in common processes in development, it is not apparent that these two pathways have a common effect on ethanol-stimulated behavior. EGFR pathway mutations affect the development of the adult brain and can suppress both the morphological and behavioral phenotypes of *tao^EP1455^*, an allele for which behavioral deficits are linked to impaired brain development [32]. In addition, we have shown previously that Egfr overexpression during development alters ethanol induced sedation behavior [31], consistent with the idea that Egfr signaling mediates the development of brain regions vital to ethanol-induced behaviors. In contrast, overexpression of *htl* can affect the adult nervous system acutely, and a *htl* allele affecting behavior shows no obvious morphological defects in the central brain. Because of technical limitations we can neither rule out a developmental role for FGFR signaling, nor an acute role for EGFR signaling in these behaviors. However, the simplest view is that these RTKs have independent effects on ethanol-stimulated behavior.

Our results raise the possibility that a conserved pathway involving *Egfr*, *tao* and *bsk* might mediate development of the MB. We observed overgrowth and midline crossing in MB β lobes in adult flies mutant for the strong *Egfr* ligand *spi*, and we observed that mutations in the weaker ligand *vn* or in the ligand-processing protease *rho* were able to suppress the MB development phenotype of *tao^EP1455^*. Further, mutations in the JNK pathway enhance genetic interactions between the EGFR pathway and *tao*, consistent with *Egfr* and *bsk* inhibiting axon outgrowth and *tao* promoting it. This mirrors the relationship between these genes that has been seen in mammalian cells: stimulation by EGF can activate the JNK pathway via small GTPases [48]–[51], and this activation can be inhibited by the Tao family kinase Taok3 [35]. Taok3 also inhibits activation of JNK in the mouse brain, modulating ethanol-induced behavior [33].

Further studies in flies carrying MB neuron clones of *Egfr, tao* and/or *bsk* mutants will be necessary to resolve their precise role of MB development. The β lobe overextension observed in *spi^1^/+* flies suggests that EGFR signaling may act predominantly in axon extension, though MB axon phenotypes in *tao^EP1455^* suggest that the defect in that mutant is in axon guidance rather than extension [32]. *Egfr* may act in conjunction with *bsk* to antagonize *tao* activity in either or both of these roles. JNK signaling both stabilizes axons of developing MB neurons and prevents their overextension [42], and interactions between *Egfr*, and the JNK pathway might occur at multiple points in MB development. Finally, it cannot be ruled out that these interactions are MB non-autonomous –they may be the result of interactions between growing MB neurons or between neurons and glia, with each pathway playing one or more roles in multiple cells.

While the MB are involved in ethanol-induced locomotion [32], it is likely that the effects of the EGFR pathway on this behavior are independent of the MB. Both gain and loss of *Egfr* function affect ethanol-induced locomotion without visible defects in MB morphology (data not shown), and *vn^1^*/+ mutants exhibit full recovery of the behavioral phenotype of *tao^EP1455^* while only partially rescuing MB morphology defects of the *tao* mutation (Fig. 2C, Fig. 4C,D,F,J). We have shown previously that a mutation in *bsk* can also fully suppress the behavioral phenotype of *tao^EP1455^* without rescuing MB morphology [33], suggesting that the behavioral phenotype of *tao^EP1455^* is not primarily due to defects in the MB [33]. As has been seen in previous studies, ethanol-induced behavior is governed by complex circuitry, and behavioral outcomes are likely the product of interactions between multiple loci [52].

It is likely, then, that *Egfr* activity is active at loci other than the MB that also mediate normal ethanol-induced locomotion. We evaluated two candidate loci in this study: dopaminergic neurons, which are part of the circuitry controlling ethanol-induced locomotion [43], and which are a locus of Egfr activity in ethanol-induced sedation behavior [10], and the EB R2/R4 neurons, which mediate ethanol-induced locomotion. However, we could not discern an effect of Egfr overexpression in either of these loci, suggesting that these neurons are not critical for the effects of *Egfr* on this behavior. Many other loci are candidate sites for EGFR activity – for instance, *aru* regulates synaptogenesis in PDF-expressing neurons that regulate circadian activity, and might be a site of EGFR activity in the developing nervous system [31]. Further experiments will be necessary to define the circuitry underlying ethanol-induced locomotion and the role of Egfr signaling in its development and function.

## Materials and Methods

### Drosophila Strains

Strains were acquired from the Bloomington Drosophila Stock Center, and from the Harvard-Exilixis Collection. The *UAS-EgfrRNAi* line (v43267) was obtained from the Vienna Drosophila RNAi Center. *The rho^iks^* strain was generously provided by Tim Tully. All mutations and transgenic lines were backcrossed into the *w^1118^* Berlin background for at least 5 generations. The *Egfr^f24^* and *vn^1^* strains, which lack positively selectable markers, were backcrossed for 5 generations to strains carrying closely linked P-element insertions that had themselves been backcrossed to w- Berlin for more than 5 generations, selecting against the P-element in each cross.

### Behavior

Ethanol-induced locomotion assays were performed as described [14], [32], using the *w-* Berlin parent strain as a control. Groups of 20 male flies were equilibrated to the test chamber and filmed using Adobe Premier during exposure to ethanol. Flies were exposed to a humidified airstream for 2 minutes, then to ethanol vapor mixed with humidified air at a ratio of 1∶2 for 21 minutes. A modified version of DIAS motion tracking software was used to measure average speed of the population at 14 timepoints over 23 minutes, including timepoints during air-only exposure to measure baseline locomotion, and during the initial olfactory startle to measure peak locomotion. Area-under-curve was calculated for resulting locomotion traces for quantification and statistical analysis. Where single comparisons were made, significance of results was assessed using Student’s T-Test. Where multiple comparisons were made, One-Way ANOVA with Neuman-Keuls post hoc test was used. All experiments represent *n* of at least 8.

### Ethanol Absorption

Groups of 20 male flies were exposed to ethanol vapor mixed with humidified air at a ratio of 2∶1 for 15 minutes (*rho^iks^*) or 20 minutes. (*Egfr^f24^/+* and *htl^d07110^/+*). Flies were frozen in liquid nitrogen, stored in −80°C and ethanol content was measured using Genzyme Diagnostics ethanol quantification kit. Whole body extracts were compared to a standard range of 0 mM to 20 mM ethanol, and single fly ethanol concentrations were calculated with the assumption that 1 fly displaces 1 uL of solution.

### Immunohistochemistry

Anti-Fas II immunohistochemistry was performed as described previously [32]. Dissected brains were fixed in PBS with 4% formaldehyde, washed with PBS with 0.3% Triton X-100, and blocked in PBS with 5% normal goat serum and 0.3% Triton X-100. Brains were incubated 24–48 hrs with anti-Fas II (Developmental Studies Hybridoma Bank) diluted 1∶200 in blocking buffer. After washing in PBS, brains were incubated with Alexa-Fluor 488 conjugated goat anti-mouse secondary antibody (Invitrogen Molecular Probes) diluted 1∶500 in blocking buffer for 1 hour. After further washing, samples were mounted using ProLong Gold (Invitrogen). Scoring of MB phenotypes was done blind to genotype. All experiments represent *n* of at least 10.

### RT-PCR

Total RNA was isolated from fly heads using Trizol reagent (Invitrogen). First strand cDNA was synthesized using the Superscript III Reverse Transcriptase kit (Invitrogen), primed with random hexamers. PCR was performed with a forward primer specific to the RB transcript of *htl* (5′-TCCAACGCAGAGACACTTTG-3′), and with a forward primer that amplifies both the RA and RC transcripts (5′-TGGCTCCGTAAAAATTCACA-3′), paired with a common reverse primer (5′-CCTTGGATCGCTTTTGATGT-3′). Primeres amplifying *RPL32* were used as a control (5′-CCAGTCGGATCGATATGCTAA-3′, 5′-GTTCGATCCGTAACCGATGT-3′). Aliquots were removed from reactions after 30, 33 and 35 cycles of amplification to verify the linear range for the assay.

## References

[1] Schuckit MA (2009) An overview of genetic influences in alcoholism. J Subst Abuse Treat 36: S5–14. 19062348

[2] Gelernter J, Kranzler HR (2009) Genetics of alcohol dependence. Hum Genet 126: 91–99 10.1007/s00439-009-0701-2 19533172 PMC3773848

[3] Sullivan PF, Daly MJ, O’Donovan M (2012) Genetic architectures of psychiatric disorders: the emerging picture and its implications. Nature Reviews Genetics 13: 537–551 10.1038/nrg3240 PMC4110909 22777127

[4] Wolf FW, Heberlein U (2003) Invertebrate models of drug abuse. Journal of Neurobiology 54: 161–178 10.1002/neu.10166 12486703

[5] Rodan AR, Rothenfluh A (2010) The Genetics of Behavioral Alcohol Responses in Drosophila. In: Matthew T Reilly and David M Lovinger, editor. International Review of Neurobiology. Academic Press, Vol. Volume 91. 25–51. Available: http://www.sciencedirect.com/science/article/pii/S0074774210910027. Accessed 27 June 2013. 10.1016/S0074-7742(10)91002-7 PMC3531558 20813239

[6] Kaun KR, Devineni AV, Heberlein U (2012) Drosophila melanogaster as a model to study drug addiction. Hum Genet 131: 959–975 10.1007/s00439-012-1146-6 22350798 PMC3351628

[7] Scholz H, Mustard JA (2013) Invertebrate Models of Alcoholism. In: Sommer WH, Spanagel R, editors. Behavioral Neurobiology of Alcohol Addiction. Current Topics in Behavioral Neurosciences. Springer Berlin Heidelberg. 433–457. Available: http://link.springer.com/chapter/10.1007/7854_2011_128. Accessed 27 June 2013.

[8] Reaume CJ, Sokolowski MB (2006) The nature of Drosophila melanogaster. Current Biology 16: R623–R628 10.1016/j.cub.2006.07.042 16920605

[9] Moore MS, DeZazzo J, Luk AY, Tully T, Singh CM, et al (1998) Ethanol Intoxication in Drosophila: Genetic and Pharmacological Evidence for Regulation by the cAMP Signaling Pathway. Cell 93: 997–1007 10.1016/S0092-8674(00)81205-2 9635429

[10] Corl AB, Berger KH, Ophir-Shohat G, Gesch J, Simms JA, et al (2009) Happyhour, a Ste20 Family Kinase, Implicates EGFR Signaling in Ethanol-Induced Behaviors. Cell 137: 949–960 10.1016/j.cell.2009.03.020 19464045

[11] Heberlein U, Tsai LT-Y, Kapfhamer D, Lasek AW (2009) Drosophila, a genetic model system to study cocaine-related behaviors: A review with focus on LIM-only proteins. Neuropharmacology 56 Supplement 197–106 10.1016/j.neuropharm.2008.07.023 18694769 PMC2819469

[12] Lasek AW, Lim J, Kliethermes CL, Berger KH, Joslyn G, et al (2011) An Evolutionary Conserved Role for Anaplastic Lymphoma Kinase in Behavioral Responses to Ethanol. PLoS ONE 6: e22636 10.1371/journal.pone.0022636 21799923 PMC3142173

[13] Rothenfluh A, Heberlein U (2002) Drugs, flies, and videotape: the effects of ethanol and cocaine on Drosophila locomotion. Current Opinion in Neurobiology 12: 639–645 10.1016/S0959-4388(02)00380-X 12490253

[14] Wolf FW, Rodan AR, Tsai LT-Y, Heberlein U (2002) High-Resolution Analysis of Ethanol-Induced Locomotor Stimulation in Drosophila. J Neurosci 22: 11035–11044. 12486199 10.1523/JNEUROSCI.22-24-11035.2002 PMC6758417

[15] Kaun KR, Azanchi R, Maung Z, Hirsh J, Heberlein U (2011) A Drosophila model for alcohol reward. Nature Neuroscience 14: 612–619 10.1038/nn.2805 21499254 PMC4249630

[16] Schweitzer R, Shilo B-Z (1997) A thousand and one roles for the Drosophila EGF receptor. Trends in Genetics 13: 191–196 10.1016/S0168-9525(97)01091-3 9154002

[17] Doroquez D, Rebay I (n.d.) Signal Integration During Development: Mechanisms of EGFR and Notch Pathway Function and Cross-Talk, Critical Reviews in Biochemistry and Molecular Biology, Informa Healthcare. Available: http://informahealthcare.com/doi/full/10.1080/10409230600914344?prevSearch=allfield%253A%2528rebay%2529&searchHistoryKey=. Accessed 31 October 2012. 10.1080/10409230600914344 17092823

[18] Freeman M (1996) Reiterative Use of the EGF Receptor Triggers Differentiation of All Cell Types in the Drosophila Eye. Cell 87: 651–660 10.1016/S0092-8674(00)81385-9 8929534

[19] Dumstrei K, Nassif C, Abboud G, Aryai A, Aryai A, et al (1998) EGFR signaling is required for the differentiation and maintenance of neural progenitors along the dorsal midline of the Drosophila embryonic head. Development 125: 3417–3426. 9693145 10.1242/dev.125.17.3417

[20] Huang Z, Shilo B-Z, Kunes S (1998) A Retinal Axon Fascicle Uses Spitz, an EGF Receptor Ligand, to Construct a Synaptic Cartridge in the Brain of Drosophila. Cell 95: 693–703 10.1016/S0092-8674(00)81639-6 9845371

[21] Sepp KJ, Auld VJ (2003) Reciprocal Interactions between Neurons and Glia Are Required for Drosophila Peripheral Nervous System Development. J Neurosci 23: 8221–8230. 12967983 10.1523/JNEUROSCI.23-23-08221.2003 PMC6740693

[22] Klämbt C, Glazer L, Shilo BZ (1992) breathless, a Drosophila FGF receptor homolog, is essential for migration of tracheal and specific midline glial cells. Genes Dev 6: 1668–1678 10.1101/gad.6.9.1668 1325393

[23] Beiman M, Shilo BZ, Volk T (1996) Heartless, a Drosophila FGF receptor homolog, is essential for cell migration and establishment of several mesodermal lineages. Genes Dev 10: 2993–3002 10.1101/gad.10.23.2993 8957000

[24] Srahna M, Leyssen M, Choi CM, Fradkin LG, Noordermeer JN, et al (2006) A Signaling Network for Patterning of Neuronal Connectivity in the Drosophila Brain. PLoS Biol 4: e348 10.1371/journal.pbio.0040348 17032066 PMC1592317

[25] García-Alonso L, Romani S, Jiménez F (2000) The EGF and FGF Receptors Mediate Neuroglian Function to Control Growth Cone Decisions during Sensory Axon Guidance in Drosophila. Neuron 28: 741–752 10.1016/S0896-6273(00)00150-1 11163263

[26] Simon MA (2000) Receptor Tyrosine Kinases: Specific Outcomes from General Signals. Cell 103: 13–15 10.1016/S0092-8674(00)00100-8 11051543

[27] Ghabrial A, Luschnig S, Metzstein MM, Krasnow MA (2003) Branching Morphogenesis of the Drosophila Tracheal System. Annual Review of Cell and Developmental Biology 19: 623–647 10.1146/annurev.cellbio.19.031403.160043 14570584

[28] Kristiansen LV, Velasquez E, Romani S, Baars S, Berezin V, et al (2005) Genetic analysis of an overlapping functional requirement for L1- and NCAM-type proteins during sensory axon guidance in Drosophila. Molecular and Cellular Neuroscience 28: 141–152 10.1016/j.mcn.2004.09.003 15607949

[29] Grigorian M, Mandal L, Hakimi M, Ortiz I, Hartenstein V (2011) The convergence of Notch and MAPK signaling specifies the blood progenitor fate in the Drosophila mesoderm. Developmental Biology 353: 105–118 10.1016/j.ydbio.2011.02.024 21382367 PMC3312814

[30] Fazioli F, Minichiello L, Matoska V, Castagnino P, Miki T, et al (1993) Eps8, a substrate for the epidermal growth factor receptor kinase, enhances EGF-dependent mitogenic signals. EMBO J 12: 3799–3808. 8404850 10.1002/j.1460-2075.1993.tb06058.x PMC413663

[31] Eddison M, Guarnieri DJ, Cheng L, Liu C-H, Moffat KG, et al (2011) arouser Reveals a Role for Synapse Number in the Regulation of Ethanol Sensitivity. Neuron 70: 979–990 10.1016/j.neuron.2011.03.030 21658589

[32] King I, Tsai LT-Y, Pflanz R, Voigt A, Lee S, et al (2011) Drosophila tao Controls Mushroom Body Development and Ethanol-Stimulated Behavior through par-1. J Neurosci 31: 1139–1148 10.1523/JNEUROSCI.4416-10.2011 21248138 PMC3045818

[33] Kapfhamer D, King I, Zou ME, Lim JP, Heberlein U, et al (2012) JNK Pathway Activation Is Controlled by Tao/TAOK3 to Modulate Ethanol Sensitivity. PLoS ONE 7: e50594 10.1371/journal.pone.0050594 23227189 PMC3515618

[34] Zhu MY, Wilson R, Leptin M (2005) A Screen for Genes That Influence Fibroblast Growth Factor Signal Transduction in Drosophila. Genetics 170: 767–777 10.1534/genetics.104.039750 15834142 PMC1450423

[35] Tassi E, Biesova Z, Fiore PPD, Gutkind JS, Wong WT (1999) Human JIK, a Novel Member of the STE20 Kinase Family That Inhibits JNK and Is Negatively Regulated by Epidermal Growth Factor. J Biol Chem 274: 33287–33295 10.1074/jbc.274.47.33287 10559204

[36] Urban S, Lee JR, Freeman M (2001) Drosophila Rhomboid-1 Defines a Family of Putative Intramembrane Serine Proteases. Cell 107: 173–182 10.1016/S0092-8674(01)00525-6 11672525

[37] Brand AH, Perrimon N (1993) Targeted gene expression as a means of altering cell fates and generating dominant phenotypes. Development 118: 401–415. 8223268 10.1242/dev.118.2.401

[38] Lin DM, Goodman CS (1994) Ectopic and increased expression of fasciclin II alters motoneuron growth cone guidance. Neuron 13: 507–523 10.1016/0896-6273(94)90022-1 7917288

[39] Lesokhin AM, Yu S-Y, Katz J, Baker NE (1999) Several Levels of EGF Receptor Signaling during Photoreceptor Specification in Wild-Type, Ellipse,and Null Mutant Drosophila. Developmental Biology 205: 129–144 10.1006/dbio.1998.9121 9882502

[40] Lanoue BR, Gordon MD, Battye R, Jacobs JR (2000) Genetic analysis of vein function in the Drosophila embryonic nervous system. Genome 43: 564–573 10.1139/g00-014 10902722

[41] Cheng Y, Endo K, Wu K, Rodan AR, Heberlein U, et al (2001) Drosophila fasciclinII Is Required for the Formation of Odor Memories and for Normal Sensitivity to Alcohol. Cell 105: 757–768 10.1016/S0092-8674(01)00386-5 11440718

[42] Rallis A, Moore C, Ng J (2010) Signal strength and signal duration define two distinct aspects of JNK-regulated axon stability. Developmental Biology 339: 65–77 10.1016/j.ydbio.2009.12.016 20035736 PMC2845820

[43] Kong EC, Woo K, Li H, Lebestky T, Mayer N, et al (2010) A Pair of Dopamine Neurons Target the D1-Like Dopamine Receptor DopR in the Central Complex to Promote Ethanol-Stimulated Locomotion in Drosophila. PLoS ONE 5: e9954 10.1371/journal.pone.0009954 20376353 PMC2848596

[44] Thibault ST, Singer MA, Miyazaki WY, Milash B, Dompe NA, et al (2004) A complementary transposon tool kit for Drosophila melanogaster using P and piggyBac. Nature Genetics 36: 283–287 10.1038/ng1314 14981521

[45] Tan Y, Yu D, Busto GU, Wilson C, Davis RL (2013) Wnt Signaling Is Required for Long-Term Memory Formation. Cell Reports 4: 1082–1089 10.1016/j.celrep.2013.08.007 24035392 PMC4083693

[46] Michelson AM, Gisselbrecht S, Zhou Y, Baek K-H, Buff EM (1998) Dual functions of the heartless fibroblast growth factor receptor in development of the Drosophila embryonic mesoderm. Developmental Genetics 22: 212–229 doi:;–10.1002/(SICI)1520-6408(1998)22:3<212::AID-DVG4>3.0.CO;2–9 9621429 10.1002/(SICI)1520-6408(1998)22:3<212::AID-DVG4>3.0.CO;2-9

[47] McGuire SE, Mao Z, Davis RL (2004) Spatiotemporal Gene Expression Targeting with the TARGET and Gene-Switch Systems in Drosophila. Sci STKE 2004: pl6 10.1126/stke.2202004pl6 14970377

[48] Coso OA, Chiariello M, Yu J-C, Teramoto H, Crespo P, et al (1995) The small GTP-binding proteins Rac1 and Cdc42regulate the activity of the JNK/SAPK signaling pathway. Cell 81: 1137–1146 10.1016/S0092-8674(05)80018-2 7600581

[49] Logan SK, Falasca M, Hu P, Schlessinger J (1997) Phosphatidylinositol 3-kinase mediates epidermal growth factor-induced activation of the c-Jun N-terminal kinase signaling pathway. Mol Cell Biol 17: 5784–5790. 9315636 10.1128/mcb.17.10.5784 PMC232426

[50] Antonyak MA, Moscatello DK, Wong AJ (1998) Constitutive Activation of c-Jun N-terminal Kinase by a Mutant Epidermal Growth Factor Receptor. J Biol Chem 273: 2817–2822 10.1074/jbc.273.5.2817 9446590

[51] Minden A, Lin A, Claret F-X, Abo A, Karin M (1995) Selective activation of the JNK signaling cascadeand c-Jun transcriptional activity by the small GTPases Rac and Cdc42Hs. Cell 81: 1147–1157 10.1016/S0092-8674(05)80019-4 7600582

[52] Rodan AR, Kiger JA, Heberlein U (2002) Functional Dissection of Neuroanatomical Loci Regulating Ethanol Sensitivity in Drosophila. J Neurosci 22: 9490–9501. 12417673 10.1523/JNEUROSCI.22-21-09490.2002 PMC6758036

